# Impact of Sample Preparation Strategies on the Quantitative Accuracy of Low-Abundance Serum Proteins in Shotgun Proteomics

**DOI:** 10.1021/acs.jproteome.5c00572

**Published:** 2025-08-26

**Authors:** Drosos Katsavelis, Marieke G. C. van der Hart, Justina C. Wolters, Hjalmar P. Permentier, Peter Horvatovich, Thomas I.F.H. Cremers

**Affiliations:** † Department of Analytical Biochemistry, 3647 University of Groningen, Antonius Deusinglaan 1, 9713 AV Groningen, The Netherlands; ‡ Quantall B.V., L.J. Zielstraweg 1, 9713 GX Groningen, The Netherlands

**Keywords:** serum proteomics, sample preparation methods, quantitative accuracy, shotgun analysis, depth of proteome, reproducibility

## Abstract

Serum proteomics plays a crucial role in biomarker discovery and disease research, yet the selection of an optimal sample preparation method remains challenging. Evaluating the accuracy of protein quantitation is of major importance and a vital part of a benchmarking study in proteomics, since clinical studies are based on the differential expression of certain proteins that can be used as biomarkers or be indicative of a pathological state. In this study, we performed a direct comparison of 6 widely used serum proteomic sample preparation workflows: In-gel digestion (IGD), Single-Pot Solid-Phase-enhanced Sample Preparation (SP3), Top 14 Abundant Protein Depletion (Top 14), Isopropanol/Trichloroacetic Acid (IPA/TCA) precipitation, PreOmics ENRICH-iST (PreOmics), and Seer Proteograph XT (Seer). Seer and PreOmics demonstrated superior quantitative accuracy, especially for proteins with low abundance in serum, while the Seer enrichment approach provided the highest number of protein identifications (>2000) as measured by an Orbitrap Exploris 480. All methods had median CVs close to or below 20%. This comparative analysis provides a comprehensive resource for selecting the most appropriate serum sample preparation strategy based on specific experimental needs, facilitating human serum proteomic profiling for biomedical research.

## Graphical abstract

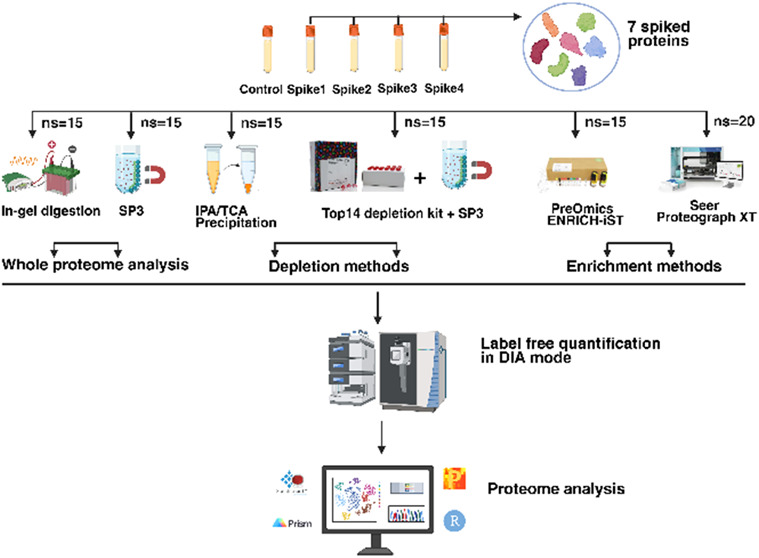

## Introduction

Proteome analysis offers a direct window into the functional state of biological systems by measuring the actual protein content, including post-translational modifications.^1^ Protein abundance reflects cellular activity, enabling the identification of disease-associated biomarkers, therapeutic targets, and dynamic responses to physiological or pathological stimuli.^2^ Therefore, proteomics serves as a powerful tool for understanding complex biological processes and advancing precision medicine. Nowadays, high-throughput protein analysis is heavily dependent on liquid chromatography coupled to mass spectrometry (LC–MS) analytical platforms. Thanks to the high analytical sensitivity, large dynamic measurement range, and specificity of this method, it is possible to detect changes in protein levels and associate these changes with various diseases.^3^ Affinity-based approaches, such as those of Olink and SomaLogic, can also be used complementarily to achieve a more comprehensive and accurate understanding of the proteome. Olink relies on proximity extension assays (PEA) using antibody pairs, while SomaLogic uses aptamer-based recognition to bind target proteins.^4,5^ However, these methods provide only protein abundance data but no information on isoforms and PTMs (post-translational modifications), and they highly depend on the availability and quality of antibodies or aptamers.

In bottom-up shotgun proteomics, proteins are first digested into peptides, most commonly by trypsin, and then peptides are analyzed by LC–MS­(/MS). This approach allows for the quantification of proteins based on the abundance of identified peptides.^6^ Recent advances, such as the development of data-independent acquisition (DIA),^7^ allow for obtaining comprehensive quantitative nonfragmented (MS1) and fragmented (MS2) peptide profiles from a sample. DIA data are highly complex and contain MS2 spectra mixed from multiple coeluting precursors, but provide deep protein coverage compared to the older data-dependent acquisition (DDA) approach, which selects for fragmentation of the most abundant precursors and misses valuable information on low-abundance peptides coeluting together with the more abundant ones.^8^

Blood is considered to be the source of choice for clinical proteomics, since it can be sampled with the minimal invasive method, it circulates through the whole body, and it contains leakage components from all tissues and organs reflecting their functional and physiological status.^9^ Most proteomics studies using blood as a starting material show a preference on plasma over serum, as the latter generally exhibits higher variation due to the different clotting methods as well as due to variations in clotting time of collected blood in clinical operational environment.^10,11^ However, proteomics analysis of both serum and plasma samples presents several challenges due to the complexity of the matrix and the large dynamic range of the protein concentration in these biofluids.^12^

More specifically, the detection sensitivity is significantly reduced because the dynamic range of even the most advanced MS instruments is not wide enough to detect protein concentrations that can be even greater than 10 orders of magnitude.^13–15^ In fact, the 22 most abundant proteins represent approximately 99% of the total protein mass in human plasma/serum, with albumin alone making up >50%.^13–15^ These high-abundance proteins overshadow signals from low-abundance ones, leading to ion suppression and pushing out proteins from the large measured dynamic intensity range during MS analysis. The low-abundance proteins hold high clinical and biological value, making them key targets for biomarker discovery, disease monitoring, and therapeutic drug development.^16–19^ Overcoming the detection challenge is critical to unlocking the full potential of human blood proteomics in clinical diagnostics and precision medicine.

To overcome this challenge, a number of blood protein extraction methods have been developed. The most basic category includes methods aiming to analyze the whole proteome, such as the in-gel digestion,^20,21^ in which proteins are separated from contaminants, such as low-molecular-weight lipids, and digested on a gel, and SP3 (Single-Pot Solid-Phase Enhanced Sample Preparation)^22^ that uses hydrophilic and hydrophobic paramagnetic beads to capture the proteins. Affinity-based depletion strategy utilizes antibody-based affinity columns to selectively remove high-abundance proteins.^23^ Top14 Abundant Protein Depletion Resin mini columns developed by Thermo Fisher are one of the most widely used approaches of this category, enabling the depletion of the 14 most abundant serum/plasma proteins, such as albumin, IgGs, transferrin, haptoglobin, α1-antitrypsin, fibrinogen, and others. Another method is based on the precipitation of low-abundance proteins^24^ via isopropanol (IPA) with trichloroacetic acid (TCA), while albumin remains soluble, and thus, it can be removed. More recent strategies attempt to achieve enrichment of low-abundance proteins either via engineered nanoparticles with different surface chemistries (Seer Proteograph XT)^25^ or via functionalized paramagnetic beads that selectively bind and enrich low-abundance proteins, like the ENRICH-iST kit developed by PreOmics.

Since sample preparation directly determines the quality, depth, and reproducibility of proteomics data, we aimed to compare these 6 methods using human-pooled serum as starting material. The majority of previous studies focusing on methods comparison in proteomics mainly used HeLa cells or tissue as a sample matrix, which have a narrower dynamic protein concentration range,^26–30^ while the few studies being conducted with blood have primarily used plasma^29,31,32^ and do not focus on the protein quantitative accuracy. In this study, we thoroughly examined the quantitative accuracy of proteins across all methods by spiking in 7 serum proteins at various concentrations and measuring their spiked-in linear range. Additionally, we evaluated the depth of protein coverage and reproducibility by applying the same LC–MS/MS and data analysis workflow.

## Experimental Section

### Reagents, Materials, and Instrumentation

NuPage LDS-Sample buffer (4X), gel electrophoresis (SDS-PAGE) 4–12% Bis-Tris 15-wells-gel, Pierce Quantitative Colorimetric Peptide Assay, High Select Top14 Abundant Protein Depletion Resin mini columns, and Pierce HeLa Protein Digest Standard were purchased by Thermo Fisher Scientific; ammonium bicarbonate (ABC), DL-dithiothreitol (DTT), iodoacetamide (IAA), formic acid (FA), trichloroacetic acid (TCA), and sodium deoxycholate (SDC) from Sigma-Aldrich; Rapid Protein Stain Coomassie Blue from Westburg; acetonitrile (ACN), isopropanol (IPA), absolute methanol, and absolute ethanol from Biosolve; sequencing grade-modified trypsin (V5111) from Promega; ENRICH-iST Kits 8x from PreOmics; Sera-Mag Carboxylate-Modified Magnetic Beads (44152105050250 (E3) and 24152105050250 (E7)) from Cytiva; pooled human serum from TCS Biosciences Ltd.; Sapphire low retention pipet tips from Greiner; and pipet tips for gel loading from Bio-Rad.

Bravo Automated Liquid Handling Platform from Agilent and Alpaqua Magnum FLX Enhanced Universal Magnet Plate from Alpaqua were used for the binding and washing steps of the SP3 protocol, while the 16-Tube SureBeads Magnetic Rack from Bio-Rad was used for the PreOmics protocol. The digested peptides were dried with the Concentrator plus/Vacufuge plus from Eppendorf. NanoPhotometer N120 from Implen was used to measure the protein concentration for IPA/TCA and Top 14 protocols. SP100 Automation Instrument was used by Seer to perform the Proteograph XT protocol.

### Experimental Design

For the purpose of this study, 6 different protein extraction methods were tested in human-pooled serum for their efficiency, reproducibility, and quantitative accuracy by preparing 3 technical replicates for 4 spiked-in concentrations of 7 serum proteins plus 1 control group (nonspiked), resulting in 15 samples per protein extraction method. For Seer, 4 technical replicates were prepared per spiked level, resulting in 20 samples, as the specific workflow is designed for either a 20- or 40-sample setup. More specifically, we compared In-gel digestion (IGD), Single-Pot, Solid-Phase-Enhanced Sample Preparation (SP3), IPA/TCA precipitation (IPA/TCA), Top 14 Abundant Protein Depletion coupled with SP3 (Top 14), PreOmics ENRICH-iST (PreOmics), and Seer Proteograph XT (Seer) human serum sample preparation workflows. The control group consisted of human-pooled serum without any spiked-in proteins. Spiked samples numbered 1–4 included 7 proteins spiked in various concentrations (Table 1). These proteins were selected based on their abundance in human serum and based on their diverse physicochemical properties (Supporting Table S1). Reproducibility was assessed by calculating the median coefficient of variation (CV%) for protein group intensities across technical replicates, while quantitative accuracy was evaluated through linear regression analysis for each spiked protein across the different concentrations. GO term enrichment was performed with the g:SCS algorithm, which performs multiple testing correction for p-values gained from GO and pathway enrichment analysis and corresponds to an experiment-wide threshold of *a* = 0.05. The number of replicates was chosen based on common practice in method comparison studies and was sufficient to evaluate variability and quantification consistency across protocols.

**Table 1:** Concentration Ranges of the 7 Spiked-in Serum Proteins

**gene name**	**protein description**	**provider**	**control**	**spike 1** (ng/μL)	**spike 2** (ng/μL)	**spike 3** (ng/μL)	**spike 4** (ng/μL)
CRP	C-reactive protein	Sigma-Aldrich		4	2	1	0.5
S100A8	protein S100A8	ACROBiosystems		4	2	1	0.5
LYZ	lysozyme C	Abcam		2	1	0.5	0.25
PPIA	peptidyl-propyl cis–trans isomerase A	Abcam		2	1	0.5	0.25
YWHAE	14–3–3 protein epsilon	Abbexa		0.5	0.25	0.125	0.0625
GLO1	lactoylglutathione lyase	Abbexa		0.4	0.2	0.1	0.05
PRKAR1A	cAMP-dependent protein kinase type I-α regulatory subunit	Abcam		0.4	0.2	0.1	0.05

### Serum Preparation

This study used commercially available pooled human serum, which was purchased from TCS Biosciences Ltd. No human subjects were directly involved, and no personally identifiable information was used. According to the bank’s policies, all analyses were performed with proper ethics. Frozen pooled human serum was aliquoted and stored at −80 °C. First, we calculated the amount of spiked-in proteins needed to create Spike 1 for 1.2 mL serum. Purchased proteins were spiked into a common batch to form Spike 1, followed by serial 2-fold dilutions of the protein mixture in PBS to create Spikes 2 to 4. Afterward, each Spike level was added into separate tubes of 1.2 mL of pooled serum in a ratio of 1:9 to reach the desired spiked-in concentrations. In addition, we added the same amount of PBS into the control group in order to maintain the same matrix concentration under all conditions. Next, the 5 different batches were aliquoted and stored at −80 °C until they were used for the sample preparation methods.

### Sample Preparation Methods

#### In-Gel Digestion (IGD)

LDS (lithium dodecyl sulfate) buffer (4×) was diluted 4 times with 100 mM ABC to a final concentration of 1×. Serum samples were thawed just before sample preparation, and 2 μL was added to 38 μL of diluted LDS buffer. After denaturation of proteins at 70 °C for 10 min at 500 rpm, 20 μL of each sample was loaded on a SDS-PAGE gel containing 15 wells. The gel was run for 7 min at 100 V, resulting in a protein migration of ± 2 cm. Then the gel was washed and stained with Coomassie Blue. Each sample was cut into uniform gel blocks, and these blocks containing all proteins were washed 3 times with 300 μL of 70/30% ABC/ACN for 30 min at 500 rpm and subsequently for 30 min once with 300 μL of 50/50% ABC/ACN and for 5 min with 300 μL of 100% ACN to destain all gel blocks. Reduction was performed by the addition of 30 μL of 10 mM DTT in 100 mM ABC and incubation at 60 °C for 30 min, followed by alkylation with 30 μL of 55 mM iodoacetamide in 100 mM ABC and incubation at RT for 30 min in the dark. A final washing step was performed with 300 μL of 100% ACN for 30 min at 500 rpm. Thereafter, the gel blocks were digested overnight with the addition of 500 ng trypsin in 100 mM ABC at 37 °C. The next day, peptides were extracted from the gel by adding 2% FA in 30 μL 50/50% Milli-Q water/ACN, and the solution was shaken for 20 min at 500 rpm twice. Afterward, peptides were diluted with Milli-Q water to a final concentration of 0.1% FA and cleaned up by solid-phase extraction (SPE). For this step, Gracepure TM SPE C18-Aq (5141486, 50 mg/1 mL) columns were used. First, columns were conditioned with 2 × 1 mL 100% ACN with 0.1% FA, followed by equilibration with 2 × 1 mL 0.1% FA. Peptides were applied to the columns and washed with 2 × 1 mL 0.1% FA. Elution was achieved with 3 × 0.2 mL 50% ACN + 0.1% FA. Finally, the solvent was removed with the vacuum concentrator (30 °C until peptides were completely dry), and the dried peptides were subsequently dissolved in 15 μL 0.1% FA in Milli-Q water.

### Solid-Phase-Enhanced Sample Preparation (SP3)

#### Beads Preparation

About 50 μL of carboxylate-modified hydrophilic magnetic beads was combined 1:1 with hydrophobic magnetic beads (100 μL in total) in a 1.5 mL tube. After washing with a 4× volume of 100 mM ABC (400 μL), the tube was placed in a magnetic rack, and the supernatant was removed. Another washing step was followed with 5× volume of 100 mM ABC (500 μL), and beads were reconstituted in 400 μL of washing solution (12.5 μg/μL).

#### Protocol

One microliter of thawed serum samples was added to 24 μL of 100 mM ABC (1% SDC) in a 96-well plate. Reduction was performed by adding 10 μL of 20 mM DTT in 100 mM ABC (1% SDC) and incubating at 60 °C for 30 min, followed by alkylation with 10 μL of 60 mM IAA in 100 mM ABC and incubation at RT for 30 min. Twenty microliter of the magnetic bead suspension was added to each well, and the plate was transferred to the Bravo AssayMap liquid handling system for performing the binding and washing steps with the use of a magnetic plate. Binding was performed by adding 65 μL of ACN to each well in order to get a final concentration of 50% ACN. This was followed by washing the samples twice with 200 μL of 80% ethanol and once with 180 μL of ACN. Beads were then resuspended in 40 μL of 500 ng trypsin in 100 mM ABC buffer solution, and overnight digestion at 37 °C was performed. Digestion was terminated by the addition of 10 μL of 5% FA in Milli-Q water to each well. After precipitation of the beads with the use of a magnetic plate, 35 μL of peptide solution was transferred to 1.5 mL tubes.

### IPA/TCA Precipitation (IPA/TCA)

Albumin depletion with isopropanol (IPA) and trichloroacetic acid (TCA) was performed as previously described.^24^ Briefly, 10 μL of serum samples were mixed with 90 μL of IPA with 1% (by volume) TCA solution. The mixture was vortexed vigorously for 2 min and then centrifuged at 1500*g* at 5 °C for 5 min. The supernatant was removed with a pipette, and the protein pellets were washed with 200 μL of methanol. The washing was accomplished by resuspending the pellets in methanol, centrifuging at 2000 rpm for 2 min, and then removing the supernatant with a pipette. Samples were sonicated for 15 min, and the protein pellets were resuspended in 100 μL of 100 mM ABC in Milli-Q water and sonicated until they were fully dissolved.

Protein concentrations were determined by measuring the absorbance at 280 nm using a nanophotometer, and digestion was accomplished by adding 500 ng of trypsin to each sample and incubating overnight at 37 °C. The next day, digestion was terminated by the addition of FA (1% final concentration). Peptides were diluted with Milli-Q water to a final FA concentration of 0.1% and cleaned with SPE, as previously described.

### Top 14 Abundant Protein Depletion Coupled with SP3 (Top 14)

Depletion of the top 14 abundant proteins was obtained with the High Select Top14 Abundant Protein Depletion Resin mini columns, according to the manufacturer’s recommendations. Briefly, 10 μL of plasma were applied to each mini column and incubated at RT with gentle end-over-end mixing for 15 min. Depleted flowthroughs were recovered by centrifugation at 1000*g* for 2 min.

Protein concentrations were determined by measuring the absorbance at 280 nm using a nanophotometer, and subsequently, 40 μg of protein was loaded onto a 96-well plate, which was then submitted to the SP3 protocol.

### PreOmics ENRICH-iST Workflow (PreOmics)

Serum samples were processed using the PreOmics ENRICH-iST Kit following the vendor’s provided instructions. In brief, EN-BEADS were placed in 1.5 mL tubes, washed 3 times with the use of a magnetic rack, and incubated with 20 μL of serum samples for 30 min at 30 °C and 1200 rpm with EN-BIND buffer. After another cycle of 3 washing steps, 50 μL of LYSE-BCT was added to each tube, and the samples were heated at 95 °C for 10 min with agitation at 1200 rpm. A trypsin/Lys C mixture was added, and the tubes were incubated at 37 °C for 2 h with shaking at 1200 rpm. The digestion process was stopped by adding the supplied stop buffer, and the remaining reaction supernatant was cleaned by using the provided filter cartridge. The peptides were eluted twice with 100 μL of elution buffer, and the eluates were combined. The peptides were then dried in a vacuum concentrator (30 °C until the peptides were completely dry) and resuspended in 15 μL of LC-LOAD buffer.

### Seer Proteograph XT Workflow (Seer)

Proteograph XT workflow was achieved with the Proteograph XT Assay Kit, which was carried out on the SP100 automation instrument by Seer based on the vendor’s instructions. Briefly, 240 μL of serum samples were aliquoted into two tubes (100 μL each), and each tube was incubated with 2 nanoparticle mixtures to create 2 fractions per sample (NPA and NPB). The remaining 40 μL of serum samples were retained for a neat serum digestion workflow comparison. After a one h incubation with NP suspensions, NP-bound proteins were captured using magnetic isolation. A series of gentle washes removed nonspecific and weakly bound proteins. Protein coronas were then reduced, alkylated, and digested with trypsin/Lys C to generate tryptic peptides. All of the steps were performed in a single reaction directly on the NPs. The in-solution digestion mixture was then desalted, and all detergents were removed by using a mixed media filter plate and a positive pressure (MPE) system. Desalted peptides were eluted in a high-organic buffer into a deep-well collection plate and were dried down in a SpeedVac.

### Determination of Peptide Concentration

For all sample preparation methods, except for the Seer protocol, peptide concentrations were determined based on the Pierce Quantitative Colorimetric Peptide Assay, and all samples were adjusted to an estimated concentration of 300 ng/μL (150 ng/μL for Top 14). For the Seer protocol, peptide quantitation was completed on the SP100 Automation Instrument by Seer using the Pierce Fluorescent Assay Kit (p/n 23290) to determine the peptide yield from each well. The peptides were then dried and reconstituted in 0.1% FA in Milli-Q to a final concentration of 100 ng/μL by us in our lab.

### LC–MS/MS Instrumentation and Settings

Liquid chromatography–mass spectrometry (LC–MS/MS) analysis was performed using Ultimate 3000 RSLCnano (Thermo Fisher Scientific, Waltham, MA) coupled to an Orbitrap Exploris 480 mass spectrometer (Thermo Fisher Scientific, Waltham, MA). LC solvents were prepared as follows: Buffer A (0.1% FA in Milli-Q water (v/v)) and buffer B (0.1% FA in 100% ACN). For each sample, 300 ng of digested peptides was loaded onto a 5 mm × 300 μm Acclaim PepMap 100 trapping microcolumn packed with 5 μm particles (Thermo Fisher Scientific, Waltham, MA) with a flow rate of 20 μL/min to clean up the samples. Each MS analysis included only the samples from a specific sample preparation method. For the Seer enrichment method, each NP suspension was analyzed independently by LC–MS, without prior mixing. After trapping and washing for 3 min with buffer A, peptides were forward-flow eluted onto a 50 cm × 75 μm Acclaim C18 PepMap 100 nanocolumn packed with 3 μm particles (Thermo Fisher Scientific, Waltham, MA). The peptides were eluted from the column at a constant flow rate of 300 nL/min with a linear gradient: 2% buffer B in 3 min, and then buffer B was increased to 45% buffer B in 90 min. This was followed by a short gradient of 1 min from 45 to 80% of solvent B, which was maintained for 12 min, then the solvent B went back to 2% for 1 min, and re-equilibration was performed with 2% solvent B for 16 min. Two system blanks (0.1% FA in Milli-Q water) and two HeLa samples were processed in the beginning and at the end of the whole process as negative and positive controls, respectively. The analytical column was kept at a constant temperature of 40 °C during the analysis.

The MS was operated in data-independent acquisition (DIA) mode. MS1 scans were acquired at *a m*/*z* range of 400 to 1200 with a resolution of 120,000 at 200 *m*/*z*. The RF lens was set to 40%, and the normalized AGC target was set to 300%. MS survey scan was followed by MS/MS DIA scan events using the following parameters: resolution at 30,000, normalized AGC 1000%, fixed normalized collision energy at 32%, RF lens at 40%, loop control was set to 31 scans per cycle, isolation window (*m*/*z*) of 8.6, and number of scan events was set to 92. Maximum injection time and scan range mode were set to auto. Data for both MS and MS/MS scans were acquired in profile mode.

### Proteome Analysis

#### Protein Identification and Quantification

The identification of spectra was performed with a human UniprotKB reviewed Swiss-Prot database (April 2023, 20.422 entries) by the software Spectronaut 19 (Biognosys AG, Zurich, Switzerland) using the default settings. Methionine by oxidation and N-terminal acetylation were allowed as variable modifications. Carbamidomethylation on cysteine was selected as the fixed modification for all methods except for IPA/TCA precipitation, in which reduction and alkylation were not performed. Only specific cleavage by trypsin was allowed, with a maximum of two missed cleavages. For the PreOmics and Seer protocols, Lys C was selected in combination with trypsin. Identification was performed with a peptide and protein False Discovery Rate (FDR) of 1%. Only precursors that passed the precursor FDR threshold of 0.01 were considered identified and used for protein interference. Label-free quantification on the MS1 level^33^ was performed for all samples and for each method separately with the MaxLFQ algorithm that derives label-free quantities based on inter-run peptide ratios. Quantification was performed using area-under-the-curve integration of peptide precursor intensities across all samples, and protein group quantity was based on the average peptide quantity. Normalization across runs was obtained using the software’s default local normalization strategy. Samples processed with the Seer Proteograph XT consisted of two different sample sets, processed with either NPA or NPB suspension, and were analyzed together. Then, the nanoparticle with the highest data completeness per protein group was selected for protein quantification. In the case that the same number of valid protein values for both nanoparticles was obtained, then the nanoparticle with the highest median intensity was chosen per protein group.

#### Data Filtering

Normalized protein intensities were imported to Perseus (version 2.0.10.0)^34^ in order to filter for missing values. Only protein groups detected in at least 60% of all of the replicates per sample preparation method (9 out of 15 or 12 out of 20 for Seer) were selected for downstream analysis. The same filter was also applied for the analysis of the spiked-in proteins in triplicate.

#### Calculation of Endogenous Concentration of Spiked-in Proteins

In cases where the endogenous spiked-in protein had a measurable signal in the control samples, the endogenous concentration was calculated by utilizing the standard addition method,^35^ dividing the y-intercept of the regression line by the slope.

#### Statistical Analysis

Protein molecular weight and pI were calculated on the ExPASy Web site tool (https://web.expasy.org/compute_pi/) and GRAVY score on the Bioinformatics.org Web site server (https://www.bioinformatics.org/sms2/protein_gravy.html). The upset plot was generated by the R package “UpSetR”. Proteins were subjected to a gene set enrichment analysis (GSEA) performed by the web tool g:Profiler,^36^ and linear regression analysis was performed with GraphPad Prism version 8.^37^

#### Cost and Duration of Sample Preparation Methods

The overall expenses for each method were estimated as cost per sample, either based on the commercial price of the kits (PreOmics, Seer, and Top 14) or calculated by the reagent cost per sample (IGD, SP3, and IPA/TCA). The cost of one-time investments such as magnetic racks and plates as well as the costs of the LC–MS/MS system and other research infrastructure elements (liquid handling system, centrifuges, etc.) were not included in our calculations. The duration of each protocol refers to the processing time of 15–20 samples in parallel without the digestion incubation and drying of the peptides but includes the time required for peptide clean-up.

## Results and Discussion

By using aliquots of the same commercial pooled human serum and thus removing biological variation from the experimental setup, we tested the following sample preparation methods: (a) whole proteome analysis methods: IGD and SP3, (b) depletion methods: IPA/TCA and Top 14, and (c) enrichment methods: PreOmics and Seer.

### Cost-Effort Analysis

Cost and time are two crucial factors to consider before performing serum proteomics with LC–MS/MS, especially in high-throughput experiments. For this reason, we performed a cost-effort analysis for each method (Table 2). The IGD protocol was the most time-consuming, taking in total approximately 9 h without accounting for the digestion incubation and the time needed for drying the peptides. Moreover, it is a complex protocol since proteins are isolated within a gel, requiring multiple washing steps and careful handling while cutting the gel slices. On the other hand, IPA/TCA was the most time-efficient method (approximately 1.5 h), while the rest of the methods required between 4 and 4.5 h. IPA/TCA was also the most affordable sample preparation method, with SP3 and IGD having similar costs. As expected, commercial methods were more expensive, with Seer requiring the highest price per sample, followed by PreOmics and Top 14. The Seer nanoparticle method required a higher amount of starting material (200 μL in total) for an optimal yield than the other methods tested. Despite the significantly higher starting material, it resulted in the lowest median peptide yield (0.025% recovery), while SP3 obtained the highest peptide recovery (41%). Apparently, the loss of material in the case of Seer was primarily due to the loss of high-abundance proteins during the enrichment process. In addition, Seer was the only fully automated method, significantly minimizing manual handling. Although SP3 and PreOmics were carried out semimanually and fully manually, respectively, both techniques are technically compatible with full automation.

**Table 2:** Cost-Effort Analysis for Each Sample Preparation Method

**sample preparation method**	**starting material (μL)**	**median peptide yield (μg)**	**recovery (%)** ^a^	**cost per sample** ^b^	**preparation time (in hours)** ^c^	**automation** ^d^
IGD	1	18.85	31	€	9	no
SP3	1	26.9	44	€	4	semi
IPA/TCA	10	123.22	20	€	1.5	no
Top 14	10	4.95	0.8	€€	4.5	semi
PreOmics	20	17.46	1.4	€€	4	no
Seer	100 + 100	1.52	0.025	€€€	4	yes

a Assuming that 1 μL of serum contains approximately 60 μg of proteins.

b €: Low cost, €€: medium cost, €€€: high cost.

c Without accounting for digestion incubation and drying of the peptides.

d SP3 and PreOmics can be fully automated.

### Depth of Protein Coverage

After filtering the protein groups for missing values, we detected protein groups ranging from 364 to 2040 corresponding to stripped peptide IDs (without taking modifications and charge state into account) ranging from 3560 to 17,002 (Figure 1A). The number of protein groups per method refers to all unique protein groups detected in at least one replicate. The highest protein loss after filtering for missing values was observed for IGD (16.7%), followed by that for Seer (13.3%) and SP3 (12.3%). All methods quantified protein groups approximately within 6 orders of magnitude of the MS signal (Figure 1B). Seer retrieved the highest number of protein groups and peptides by far, followed by PreOmics, while IPA/TCA resulted in the lowest number of protein and peptide identifications. We suspect that the reason for the latter result is the incomplete resolubilization of the IPA/TCA protein aggregates (even after sonication), resulting in protein loss, especially of the low-abundance serum proteins, as previously reported with the TCA/acetone combination.^27^ The broader proteomic depth of Seer could be explained by the increased enrichment of low-abundance proteins and by the fact that the starting volume of serum was at least 5-fold higher than that of the other sample preparation methods tested in this paper. This could be a restricting factor for some studies, such as in the case of rodent serum proteomics, which have a limited amount of sample starting material. We also noticed that Seer achieved the most efficient removal of albumin across all methods (Supporting Table S2). Overall, 2422 unique protein groups were detected by all methods, 1335 of which were detected only by Seer (Figure 1C).

**Figure 1:**
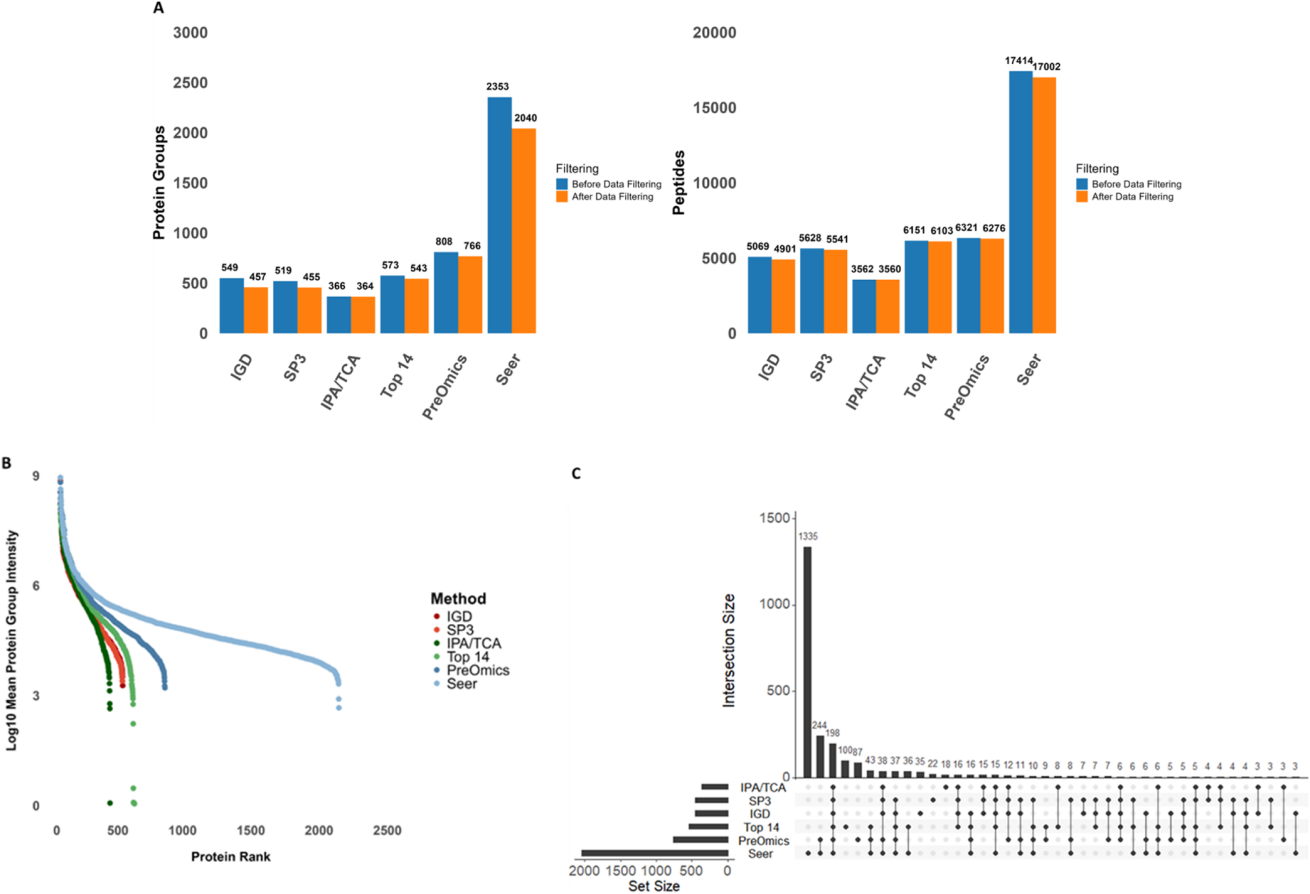
Comparison of protein and peptide detection performance in human serum. (A) Depth of protein and peptide coverage before and after filtering out entries with a high number of missing values for all sample preparation methods. (B) Mean protein group intensity for protein groups quantified by each method, sorted and ranked from most abundant to least abundant. Whole proteome analysis methods are highlighted with red color, depletion methods with green color, and enrichment methods with blue color. (C) Upset plot representing the protein group overlaps across all methods.

The total ion chromatograms (TICs) from all methods showed sharp, well-resolved peaks across the full retention time range, indicating good chromatographic separation (Supporting Figure S1). After filtering, the average missing values ranged from 1.37% for IPA/TCA to 3.58% for Seer (Supporting Figure S2).

The digestion efficiency was evaluated by calculating the percentage of missed cleavages per protein extraction protocol (Supporting Figure S2). Seer and PreOmics outperformed the rest of the methods with 88.02 and 87.57% of total peptides, respectively, without any missed cleavages. Our first thought was that the combined use of trypsin and Lys C in these methods resulted in higher proteolysis efficiency in comparison to trypsin alone in the other protocols. However, the Top 14 was also performed at a similar level (86.01%) even though proteins were digested only with trypsin. Nevertheless, the use of both enzymes would most probably enhance the digestion efficiency of all methods^38,39^ and is something that needs to be considered when comparing sample preparation methods used for serum proteomics. The highest percentage of missed cleavages (29.31% in total) was observed in IPA/TCA, most probably due to the not fully resolubilized precipitates, which can make proteins less accessible to trypsin. In addition, proteins in the IPA/TCA method were not reduced and alkylated, which could limit their unfolding and the prevention of disulfide reformation, reducing cleavage efficiency.

Different physicochemical properties may determine whether a protein is efficiently captured or lost during sample preparation. Protein hydrophobicity was measured based on the Gravy index score using the Kyte-Doolittle scale. As shown in Supporting Figure S3, the majority of the proteins from all methods tend to be slightly hydrophilic, with a median GRAVY score just below 0. The pattern of the isoelectric point of proteins was also very similar across methods, with the median values being close to 6. Top 14 and Seer were able to recover more proteins with high molecular weight than the rest of the methods.

In order to highlight how each method affects protein recovery from specific cellular regions, we performed a gene ontology (GO) analysis for the cellular component (CC) category. The top 10 gene ontology enrichments for the cellular component for each sample preparation method based on the p-adjusted values are demonstrated in Supporting Figure S4. Seer was the only method able to identify proteins related to cytoplasm, cytosol, and intracellular vesicles within the top 10 hits, since it could capture many more low-abundance proteins than the other methods.

### Reproducibility

The reproducibility of each method was assessed by the median coefficient of variation (CV%) for the protein group intensities across all replicates (Figure 2A). IPA/TCA (10.78%) outperformed the rest of the methods, having 70% of the proteins quantified with a CV lower than 20%, followed by IGD (14.96%) with 60% of proteins quantified with CVs below 20%. Top 14 (16.81%), PreOmics (17.14%), and SP3 (18.48%) quantified around 55% of their proteins with CVs below 20% while Seer exhibited the highest median CV (21.15%), with 47% of the proteins having a CV lower than 20%.

**Figure 2:**
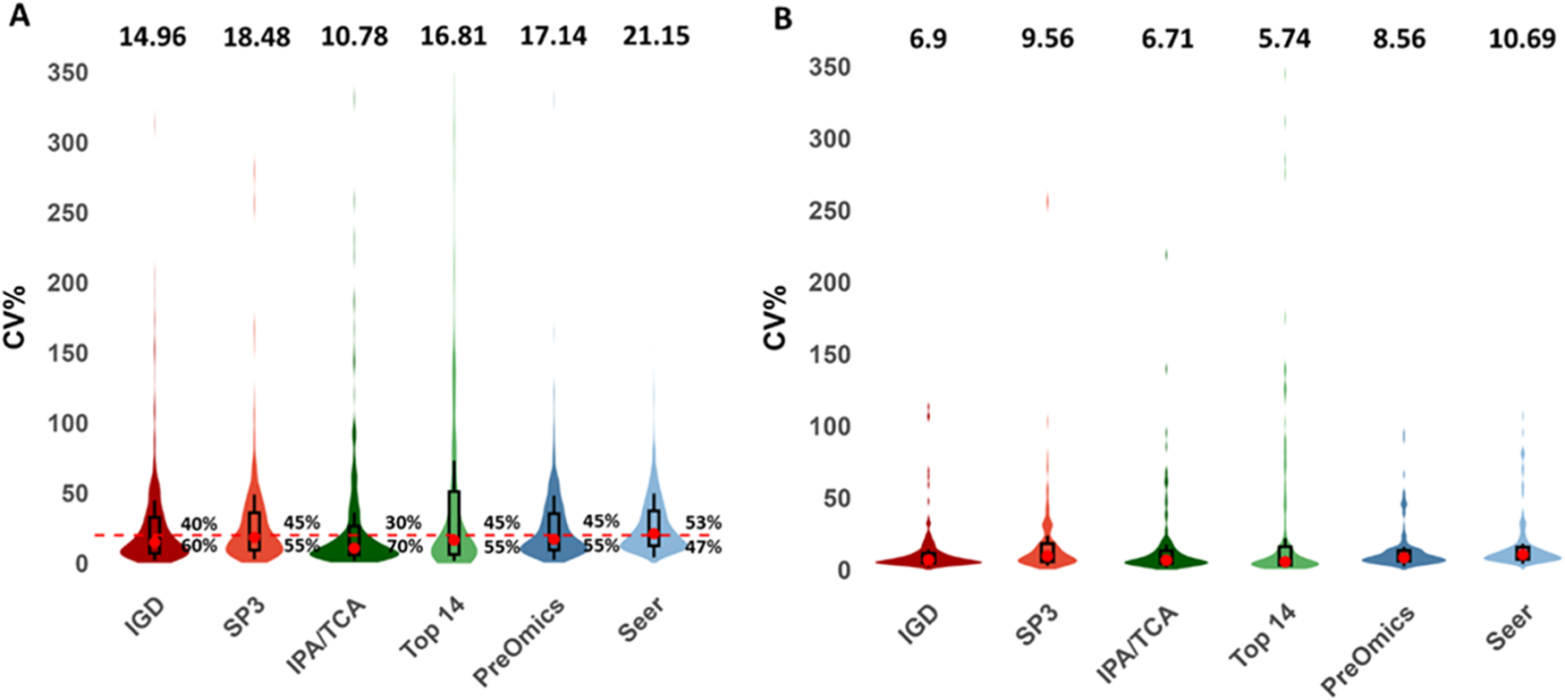
Reproducibility of human serum sample preparation methods. Whole proteome analysis methods are highlighted with red color, depletion methods with green color, and enrichment methods with blue color. (A) Distribution of the coefficient of variation (CV%) for protein group intensities across the 6 methods, with median values indicated on top and the percentage of protein groups above and below 20% CV (dashed horizontal line). (B) CV% distribution for shared protein groups (*n* = 198) across the 6 methods, with median values displayed on top. Medians and interquartile ranges in the form of a box plot are shown.

All methods had median CVs below 20% (Seer was slightly above the threshold), which is generally acceptable for most proteomics experiments, especially when working with complex biological matrices, such as serum, with an extremely wide dynamic range of the analyzed protein abundances. The very low median CV of IPA/TCA showed that there is no correlation between high variation in protein quantification and a high number of missed cleavages. We suspect that this is due to the fact that this method quantified mostly high-abundance proteins, which are known to be more tightly regulated and measured with higher signal and higher signal-to-noise ratio of their constituting peptides. Top 14 reported the most extreme CV values, mainly corresponding to immunoglobulins that were probably incompletely depleted.

Next, we wanted to see if this pattern is also conserved when focusing on the shared protein groups among all methods (*n* = 198). As expected, a major reduction in the median CV values was observed for all methods (Figure 2B). Seer, SP3, and PreOmics accounted again for the highest median values (10.69, 9.56, and 8.56%, respectively) as observed during the CV analysis of the whole proteome. Top 14 reported the lowest median CV value (5.74%), closely followed by IPA/TCA (6.71%) and IGD (6.9%).

In order to investigate the relation between protein intensity and reproducibility for the common protein groups across methods, we plotted the CV% versus log_10_ mean protein intensities (Supporting Figure S5). For this analysis, we only focused on the inherent variation caused by the sample preparation methods, and for this reason, spiked-in proteins and contaminants like keratins were excluded. The top 10 shared protein groups with the highest and lowest CVs per method are shown in Supporting File 3. Proteins from the complement system, which are generally tightly regulated in human serum, had some of the lowest CV values in all of the methods.

### Quantitative Accuracy of Spiked-in Proteins

If a sample preparation method lacks accuracy in protein quantitation, especially the quantitative accuracy of the low-abundance proteins, the measured intensities may not accurately reflect the real changes in protein abundance, leading to incorrect biological interpretations and false negatives. Thus, evaluating the accuracy of protein quantitation by sample preparation methods is of major importance.

For this reason, we spiked 7 serum proteins in different concentrations, which have different endogenous concentration abundances in human blood, and assessed their linearity. The decision on the concentration range for each spiked protein was based on their endogenous concentration in blood as stated in the Human Protein Atlas database^40^ (https://www.proteinatlas.org/humanproteome/blood) (Supporting Table S3). In the end, we divided the spiked proteins into 3 ranges: (a) high spiked-in concentrations (0.5–4.0 ng/μL for CRP and S100A8), (b) medium spiked-in concentrations (0.25–2.0 ng/μL for LYZ and PPIA), and (c) low spiked-in concentrations (0.05–0.4 ng/μL for GLO1 and PRKAR1A, and 0.0625–0.5 ng/μL for YHWAE).

Some proteins were detected in all spiked levels (control, Spikes 1–4), others in some of the spiked levels, and others were not detected at all, depending on the method used (Figure 3, Supporting Figure S6). We noticed that 1 out of the 3 IGD replicates had an outlier value for S100A8 and LYZ for the control and Spike 4 groups, respectively, and these values were excluded from the linear regression calculations. Figure 3 shows the linearity assessment of 3 spiked-in proteins from each concentration range. An overview of the endogenous concentrations of the spiked-in proteins, as measured by the standard addition method, across all methods is presented in Supporting Table S4. The standard addition method accounts for the native protein present in the sample by spiking known concentrations of the analyte into the matrix and measuring the response. By plotting the signal intensity against the amount added and extrapolating the linear regression line to the *x*-axis (where the response would be zero), the x-intercept corresponds to the negative endogenous concentration of the analyte in the unspiked sample. As a result, the total concentration ranges used for regression varied across methods depending on the amount of endogenous protein retained or lost during sample preparation. The calculated endogenous concentrations were in some cases closely aligned with the concentrations found in the Protein Atlas database for all methods, such as in the case of LYZ, while for other spiked-in proteins, such as S100A8, endogenous concentrations were more diverse across methods. Seer was the only method able to measure the endogenous signal of YWHAE and PRKAR1A (Supporting Tables S3 and S4).

**Figure 3:**
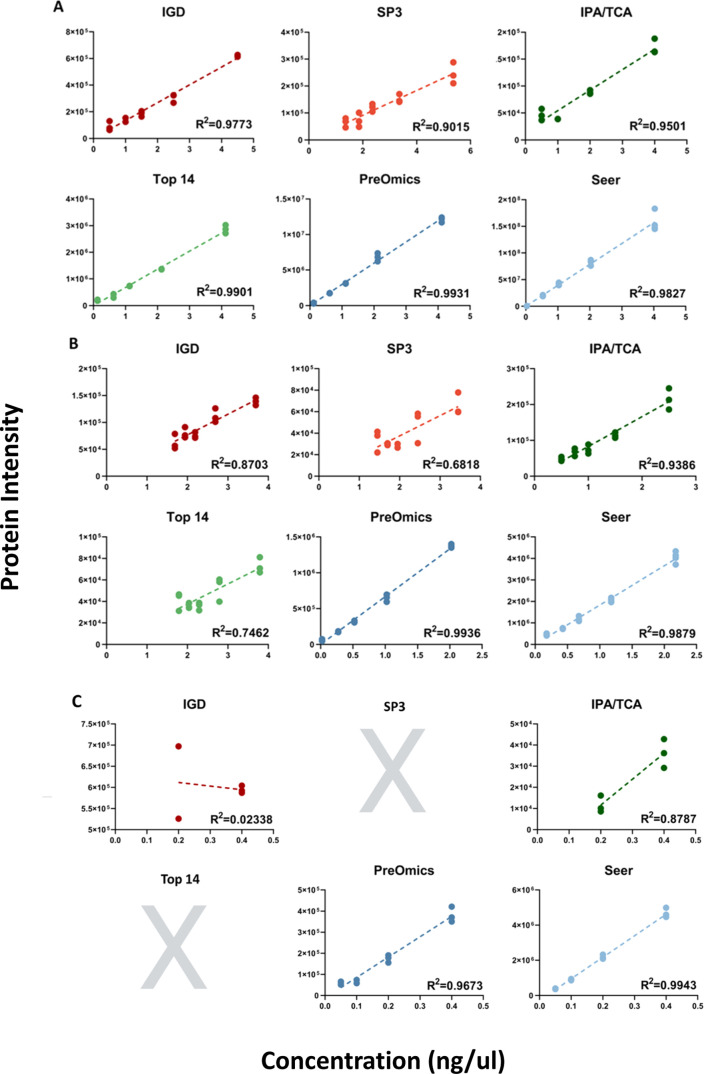
Representative examples (high, medium, and low concentration ranges) of linearity and variance explained by the linear model of spiked-in proteins across the 6 sample preparation methods by plotting the protein intensities of all replicates versus the spiked concentrations of (A) S100A8, (B) PPIA, and (C) GLO1. Whole proteome analysis methods are highlighted with red color, depletion methods with green color, and enrichment methods with blue color.

Table 3 demonstrates how well the linear model explained the variability via the calculation of *R*-squares (*R* ^2^) for all spiked-in proteins across all methods. Depending on the method used, each protein was identified by a different number of peptides (Supporting Table S5). Seer was the only method that quantified all spiked proteins, detecting the most peptides for the proteins of low spiked concentrations. To assess the precision of each sample preparation method, we calculated the average CV% for each of the 7 spiked-in proteins across the 4 concentration levels, while the standard deviation (SD) of the CVs provided an indication of how consistently that precision was maintained (Supporting File 3). Seer and PreOmics methods consistently demonstrated low average CVs and low SDs, suggesting both high precision and stability across the concentration ranges. Seer outperformed the other methods, particularly for proteins spiked-in at low concentrations (GLO1, PRKR1A, and YWHAE) by yielding the lowest average CVs and showing minimal variability across the concentration levels.

**Table 3:** Goodness of Fit Determined by Linear Regression Models for the Spiked-in Proteins

	high concentration		medium concentration		low concentration		
sample preparation method	CRP	S100A8	LYZ	PPIA	GLO1	PRKAR1A	YWHAE
IGD	0.9664	0.9773	0.9572	0.8703	0.02338	0.7431	
SP3	0.9785	0.9015	0.9142	0.6818		0.8604	
IPA/TCA	0.9852	0.9501	0.5307	0.9386	0.8787		
Top 14	0.9785	0.9901	0.9898	0.7462		0.8779	0.7563
PreOmics	0.9623	0.9931	0.9914	0.9936	0.9673	0.978	
Seer	0.9858	0.9827	0.9166	0.9879	0.9943	0.8968	0.9562

The results demonstrated that the selection of the sample preparation method plays a decisive role in the quantitative accuracy of proteins, especially for the detection of proteins in lower concentrations. However, it was still not clear if the reduced quantitative accuracy that was reported for the proteins spiked in medium and low concentrations by most of the tested sample preparation methods was exclusively due to matrix effects and method-specific limitations or if the MS reached the lower limit of detection (LOD). For this reason, we prepared a mixture of the proteins we previously spiked in serum with the same concentrations as in the Spike 1 condition, but this time the proteins were added into a 100 mM ABC buffer solution in order to avoid matrix interferences. After reduction, alkylation, and overnight digestion with trypsin, the digested proteins were diluted, but this time, a serial 2-fold dilution of the protein mixture was performed, diluting it 10 times in total. The results exhibited excellent linearity for all spiked proteins for concentrations much lower than the ones tested in serum (Supporting Figure S7 and File 3), proving that any increase of variance during the spiked-in experiment was due to effects caused by the sample preparations in combination with matrix effects, such as ion suppression, and not due to saturation of the MS detector.

## Conclusions

In summary, our findings highlight that the selection of sample preparation method in serum proteomics plays a crucial role in the final outcome, with nanoparticles technology showing the most promising results, not only in matters of depth of proteome coverage but also regarding the accuracy of quantitation, especially for low-abundance serum proteins, which carry the greatest clinical and diagnostic potential.

However, given the diversity of research goals in serum proteomics, from high-throughput biomarker discovery to focused validation studies, no single method fits all purposes. Therefore, we provide a practical overview that aligns each evaluated method with suitable research scenarios based on performance, cost, sample input, and automation potential (Table 4). This serves as a decision-making framework to guide experimental planning and aid researchers in selecting the optimal serum proteomics workflow based on their specific needs.

**Table 4:** Summary of Recommended Use Cases for Each Serum Sample Preparation Method

**sample preparation method**	**best suited for**	**strengths**	**limitations**
IGD	small-scale studies targeting high- and medium-abundance proteins	low cost; simple equipment; clean samples due to multiple washes; good quantitation for high-abundance proteins; high reproducibility; low sample input	time-consuming; low depth; manual; poor quantitation for medium- and low-abundance proteins
SP3	experiments targeting high- and medium-abundance proteins; throughput depends on the automation level	low-cost; good quantitation for high-abundance proteins; potentially fully automated; low sample input	low depth; pipet robot and instrumentation training required if automated; moderate reproducibility; poor quantitation for medium- and low-abundance proteins
IPA/TCA	targeted analysis focused on high-abundance proteins	time-efficient; low cost; simple equipment; high reproducibility	low depth; manual; samples retain impurities even after cleanup; poor quantitation for medium- and low-abundance proteins
Top 14	studies aiming to explore high- and medium-abundance proteins	time-efficient; improves access to lower abundance proteins; can be combined with numerous sample preparation methods	medium cost; depletion variability; poor quantitation for medium- and low-abundance proteins
PreOmics	studies targeting medium- to low-abundance protein quantification; throughput depends on the automation level	relatively low sample input needed; improves access to lower abundance proteins; good quantitation for high-, medium- and low-abundance proteins; ready-to-use format; potentially fully automated	medium to high cost; pipet robot and instrumentation training required if automated
Seer	high-throughput studies aiming for biomarker discovery	deep proteome coverage; good quantitation for high-, medium- and low-abundance proteins; automated workflow	high cost; pipet robot and instrumentation training required; high sample input

## Supplementary Material











## Data Availability

The mass spectrometry proteomics raw data (.raw files) as well as the files with the annotated spectra (.sne files) have been deposited to the ProteomeXchange Consortium via the PRIDE partner repository (https://www.ebi.ac.uk/pride/archive/) with the data set identifier PXD062785.

## Notes

TIC chromatograms (Figure S1), average missing values and missed cleavages (Figure S2), physicochemical properties (Figure S3), gene ontology for cellular component (Figure S4), CV versus protein group intensities (Figure S5), linearity of spiked-in proteins in serum (Figure S6), linearity of spiked- in proteins in ABC buffer solution (Figure S7); physicochemical properties of spiked-in proteins (Table S1), efficiency of albumin removal (Table S2), endogenous concentrations of spiked-in proteins (HPA) (Table S3), endogenous concentrations of spiked-in proteins (Table S4), and number of peptide IDs for spiked-in proteins (Table S5) (PDF)
Supplemental File 1: Protein groups per method (XLSX)
Supplemental File 2: Precursors per method (XLSX)
Supplemental File 3: Top 10 highest and lowest CVs per method for the shared protein groups, detailed summary of the spiked-in experiment in serum, and concentration ranges and protein intensities for the spiked-in experiment in ABC buffer solution (XLSX)
Supplemental File 4: Reports with information about protein coverage, precursor uniqueness, number of missed cleavages per precursor, precursor score, charge, and mass to charge ratio (XLSX)
