# Supplementary material for: Impact of Sample Preparation Strategies on the Quantitative Accuracy of Low-Abundance Serum Proteins in Shotgun Proteomics
Source: J Proteome Res. 2025 Aug 26;24(9):4792–803. doi: 10.1021/acs.jproteome.5c00572 (PMC12418494; doi:10.1021/acs.jproteome.5c00572)
Supplement: Supplementary file 1 [file pr5c00572_si_001.pdf]

Supporting Information

Impact of Sample Preparation Strategies on The Quantitative Accuracy of Low-Abundance Serum Proteins in Shotgun Proteomics

Drosos Katsavelis*[1,2], Marieke G.C. van der Hart[1,2], Justina C. Wolters[1], Hjalmar P. Permentier[1], Peter Horvatovich[1], Thomas I.F.H. Cremers[1,2]

[1] Department of Analytical Biochemistry, University of Groningen, Antonius Deusinglaan 1, 9713 AV Groningen, the Netherlands

[2] Quantall B.V., L.J. Zielstraweg 1, 9713 GX Groningen, the Netherlands

*Author correspondence to drosos.katsavelis@quantall.com

Table of Contents

Supplementary Figures

Supplementary Figure S1: TIC chromatograms S2
Supplementary Figure S2: Average missing values and missed cleavages S3
Supplementary Figure S3: Physicochemical properties S4
Supplementary Figure S4: Gene Ontology for Cellular Component S5
Supplementary Figure S5: CV versus protein group intensities S6
Supplementary Figure S6: Linearity of spiked-in proteins in serum S7
Supplementary Figure S7: Linearity of spiked-in proteins in ABC buffer solution S8

Supplementary Tables

Supplementary Table S1: Physicochemical properties of spiked-in proteins S9
Supplementary Table S2: Efficiency of albumin removal S9
Supplementary Table S3: Endogenous concentrations of spiked-in proteins (HPA) S10
Supplementary Table S4: Endogenous concentrations of spiked-in proteins S10
Supplementary Table S5: Number of peptide IDs for spiked-in proteins S11

Supplemental Files

Supplemental File 1: Protein groups per method (XLSX)
Supplemental File 2: Precursors per method (XLSX)
Supplemental File 3: Top 10 highest and lowest CVs per method for the shared protein groups, detailed summary of the spiked-in experiment in serum and concentration ranges with protein intensities for the spiked-in experiment in ABC buffer solution (XLSX)
Supplemental File 4: Reports with information about protein coverage, precursor uniqueness, number of missed cleavages per precursor, precursor score, charge and mass to charge ratio (XLSX)

Supplementary Figures

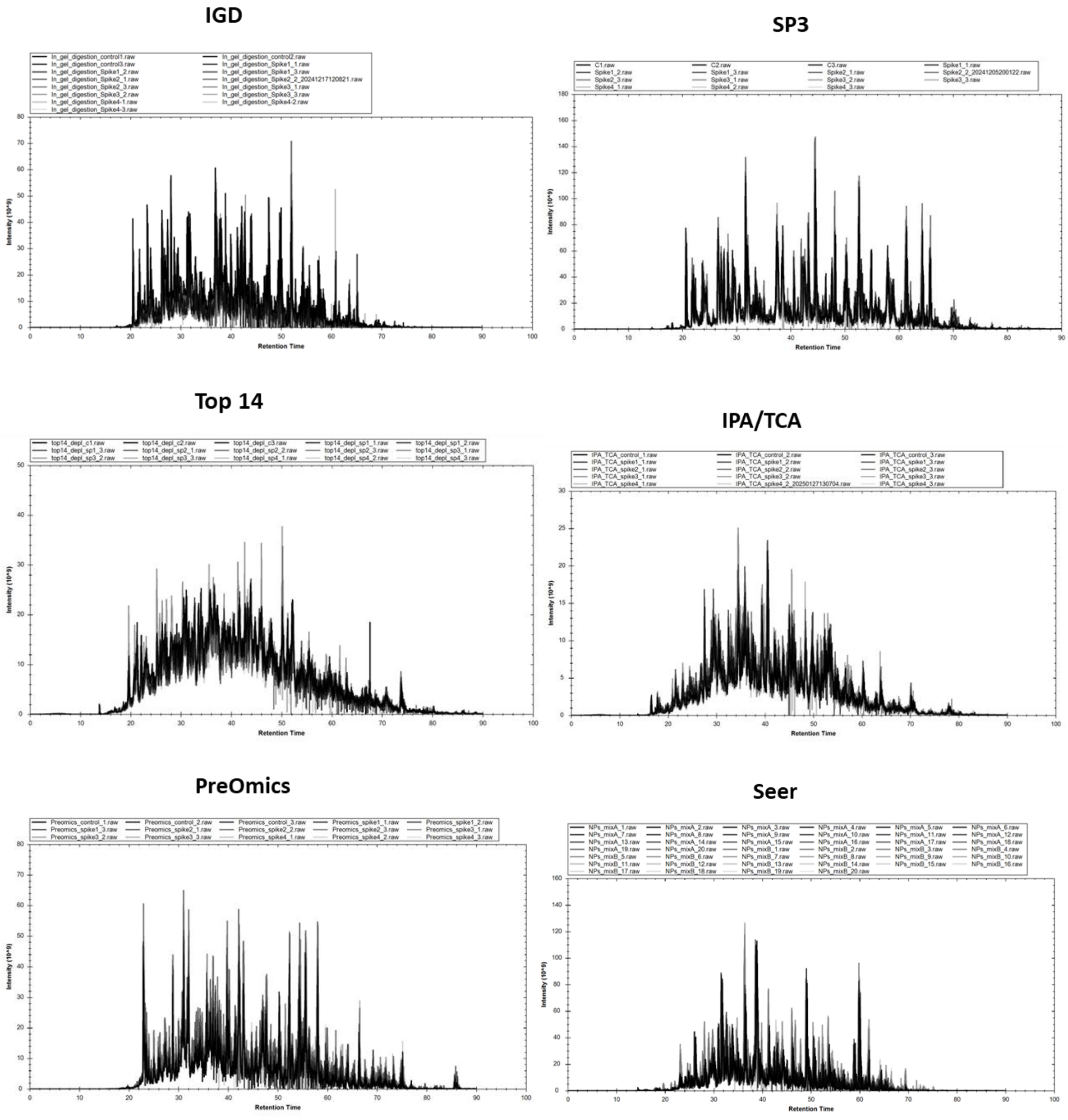

Supplementary Figure S1. Total ion current (TIC) chromatograms from all samples of each method over a 90s-minute LC gradient, as displayed in Spectronaut.

A

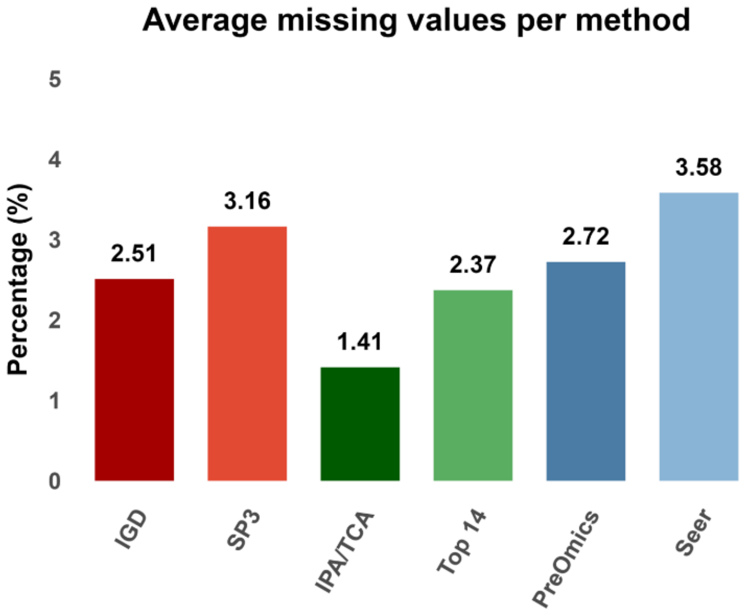

B

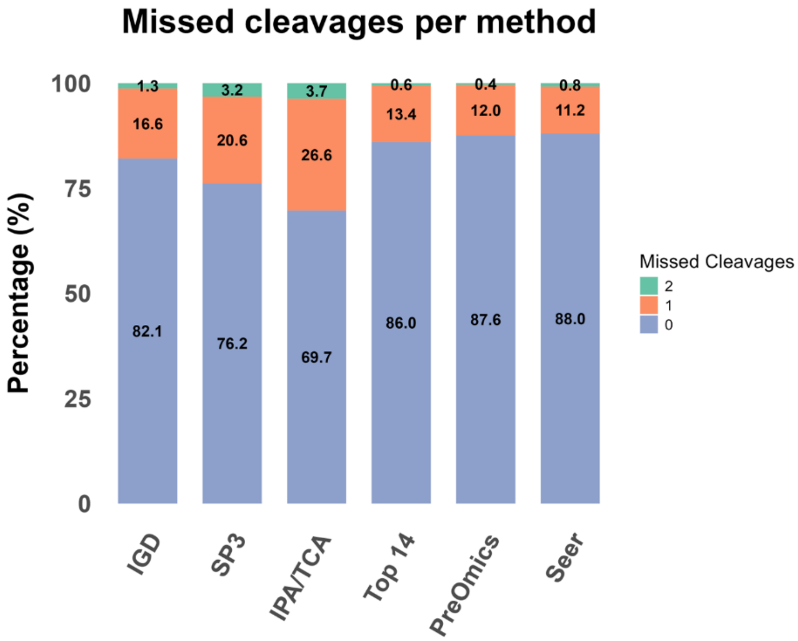

Supplementary Figure S2. A) Mean percentage of missing values after data filtering for high number of missing values per method. Whole proteome analysis methods are highlighted with red color, depletion methods with green color and enrichment methods with blue color. B) Percentage of missed cleavages across all methods.

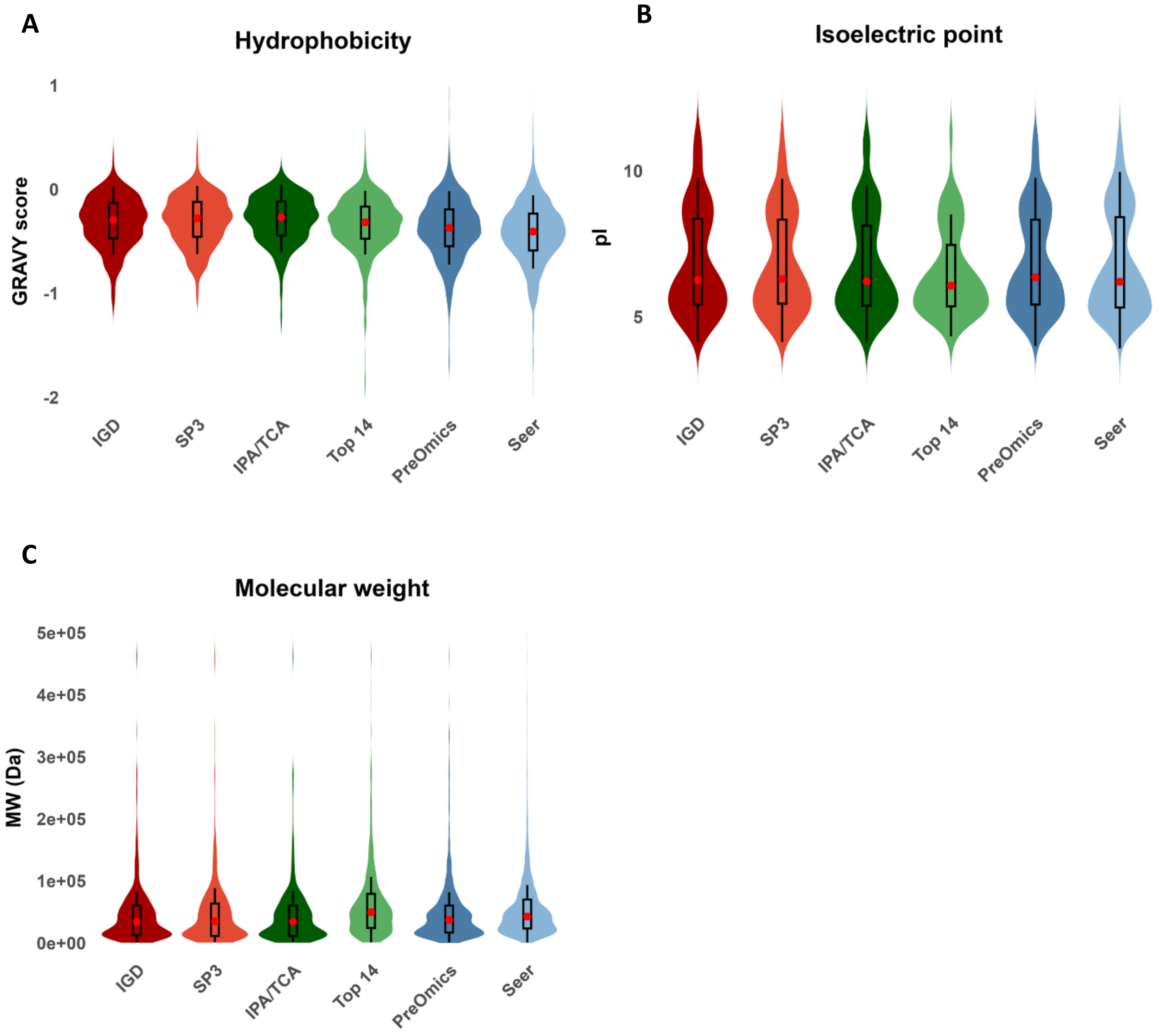

Supplementary Figure S3. Distribution of physicochemical properties of identified proteins in human serum across all methods. A) GRAVY score, B) isoelectric point and C) molecular weight. Whole proteome analysis methods are highlighted with red color, depletion methods with green color and enrichment methods with blue color. Medians and interquartile ranges in form of a box plot are shown**.**

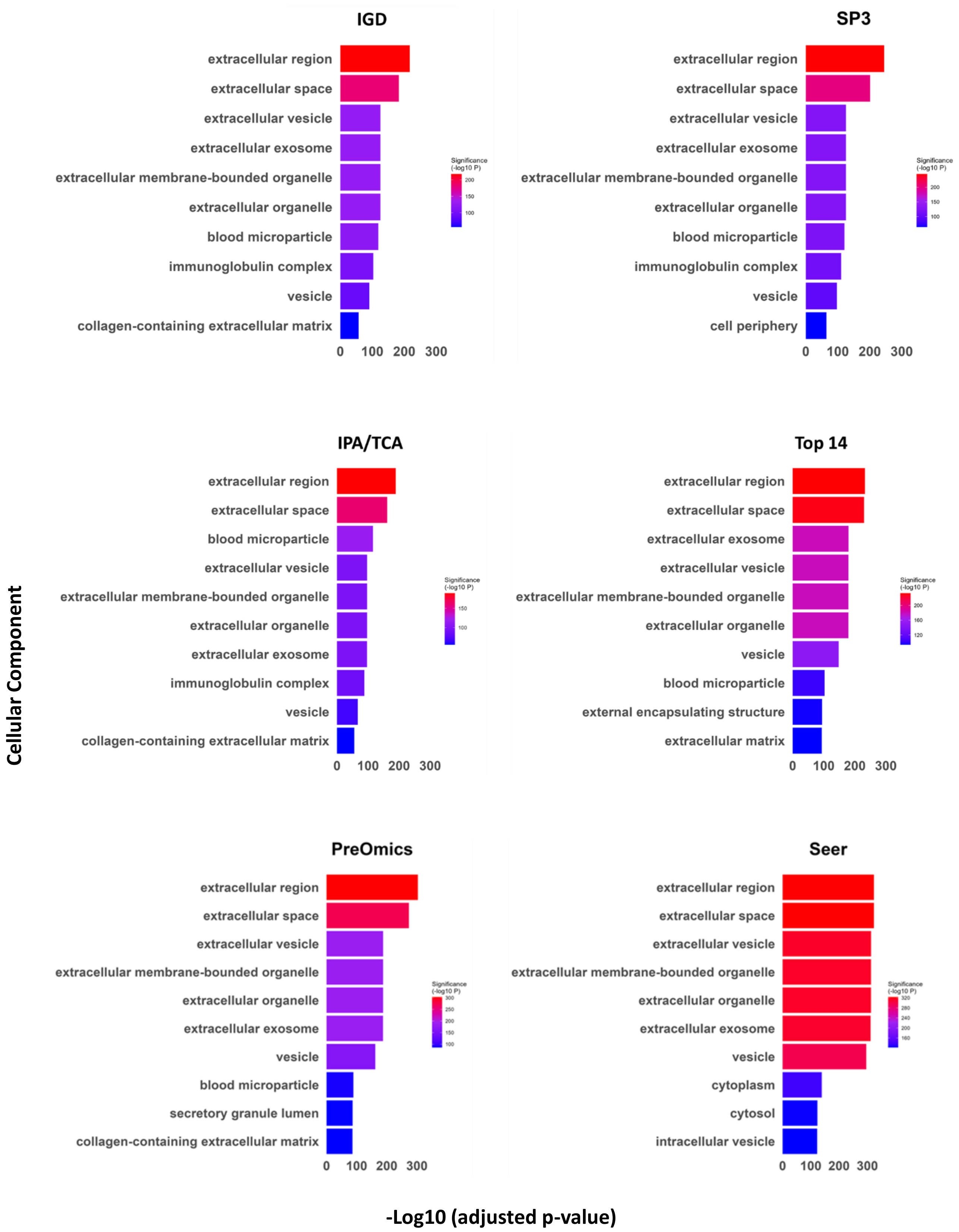

Supplementary Figure S4. Top 10 Gene Ontology enrichments for cellular component across all methods based on the adjusted p-values.

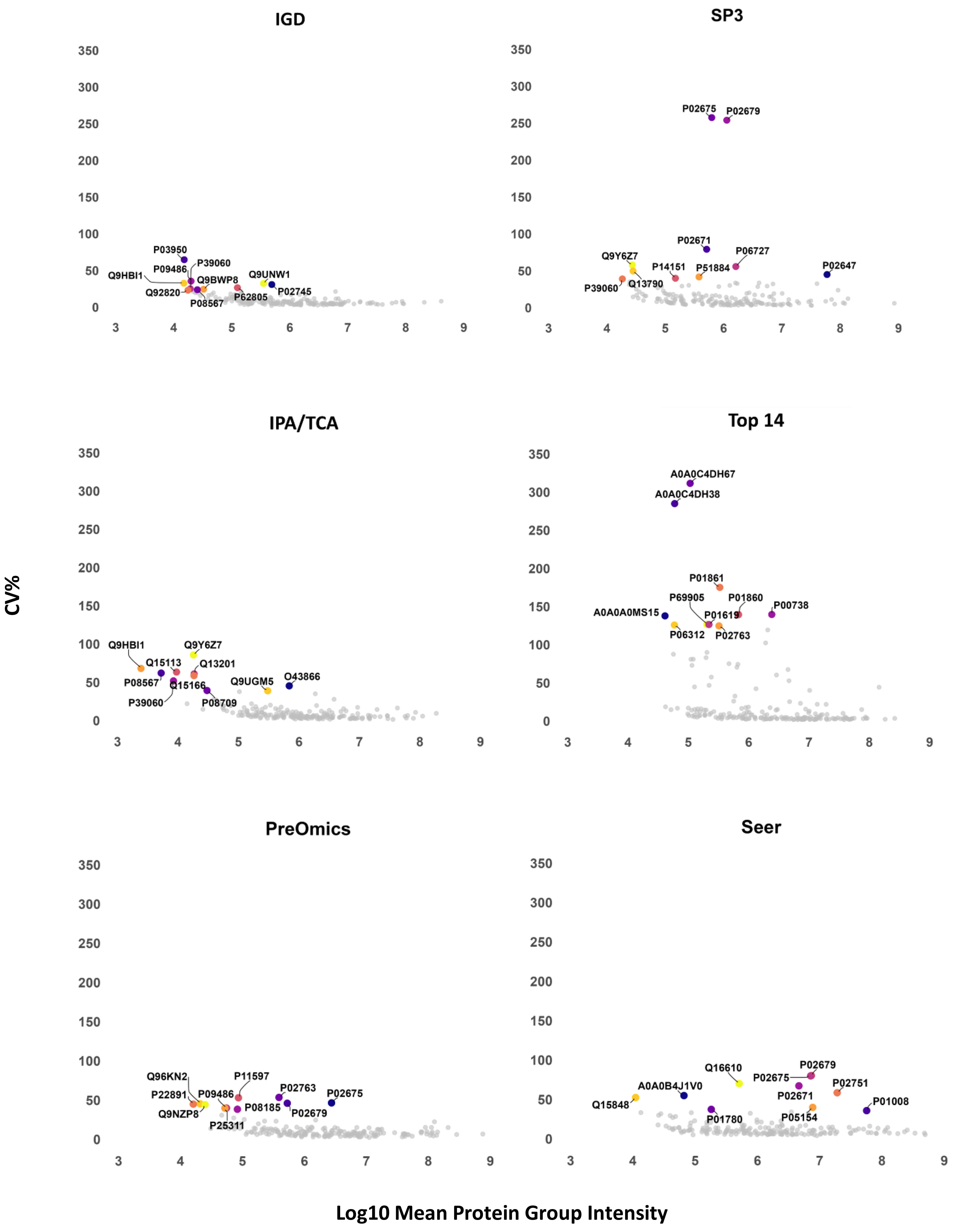

Supplementary Figure S5. Scatter plot showing the CV% and average $\log_{10}$ protein group intensity for shared proteins across all methods. The top 10 protein groups with the highest CV values are colored and labelled.

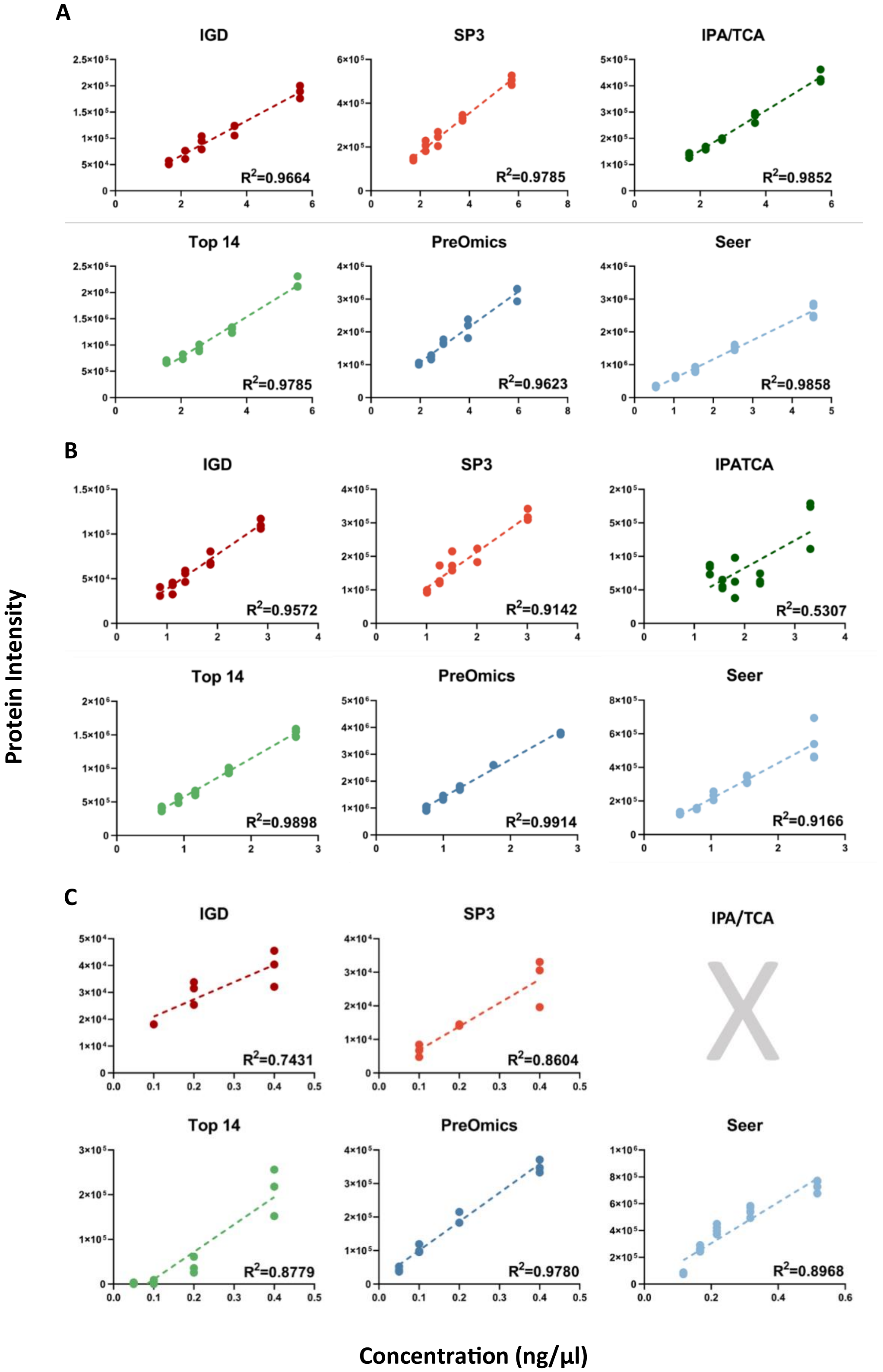
A
IGD
R2=0.9664
SP3
R2=0.9785
IPA/TCA
R2=0.9852
Top 14
R2=0.9785
PreOmics
R2=0.9623
Seer
R2=0.9858
B
IGD
R2=0.9572
SP3
R2=0.9142
IPATCA
R2=0.5307
Top 14
R2=0.9898
PreOmics
R2=0.9914
Seer
R2=0.9166
C
IGD
R2=0.7431
SP3
R2=0.8604
IPA/TCA
Top 14
R2=0.8779
PreOmics
R2=0.9780
Seer
R2=0.8968
Protein Intensity
Concentration (ng/µl)

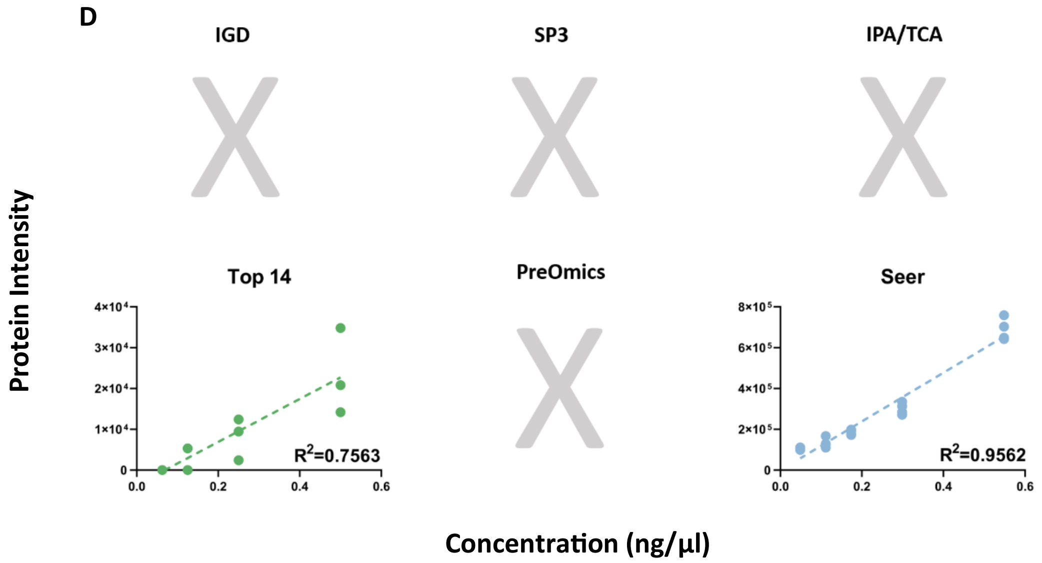

Supplementary Figure S6. Linearity and variance explained by the linear model of spiked-in proteins across the 6 sample preparation methods by plotting the protein intensities of all replicates versus the spiked concentrations of A) CRP, B) LYZ, C) PRKAR1A and D) YWHAE. Whole proteome analysis methods are highlighted with red color, depletion methods with green color and enrichment methods with blue color.

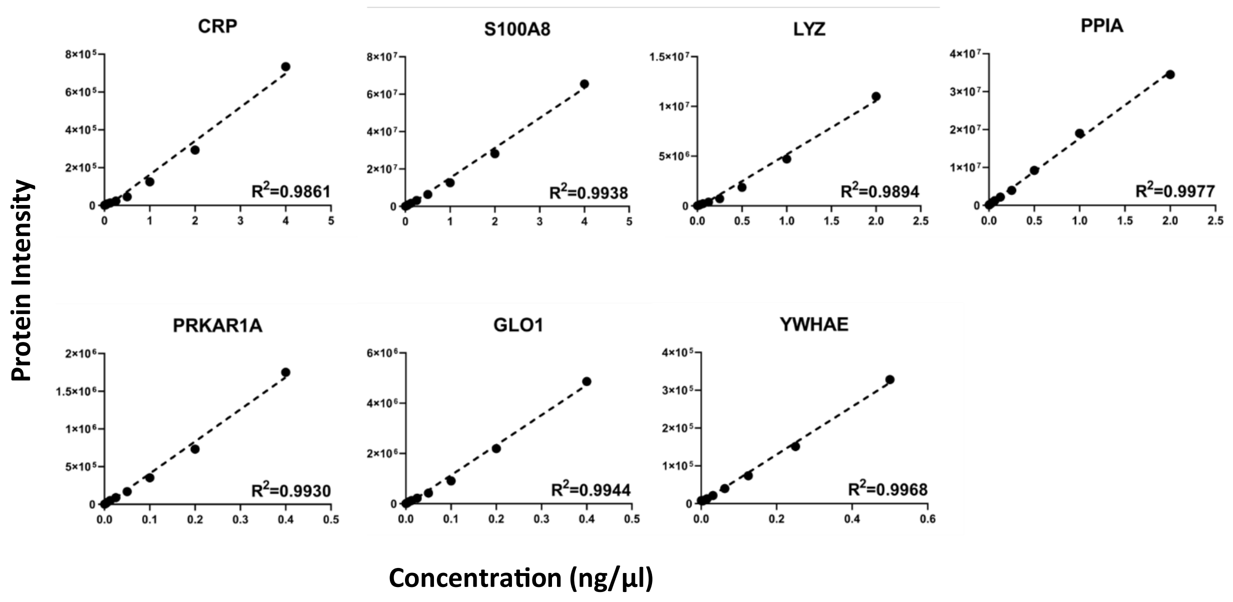

Supplementary Figure S7. Linearity assessment of the spiked-in proteins tested in 100 mM ammonium bicarbonate buffer solution.

Supplementary Tables

Supplementary Table S1. Physicochemical properties of spiked-in proteins

Gene name	Protein Description	Number of amino acids	Molecular weight (Da)	Theoretical pI	Grand average of hydropathicity (GRAVY)
CRP	C-reactive protein	206	23047.14	5.28	-0.14
S100A8	Protein S100-A8	93	10834.51	6.5	-0.397
LYZ	Lysozyme C	130	14700.67	9.28	-0.485
PPIA	Peptidyl-prolyl cis-trans isomerase A	165	18012.49	7.68	-0.316
YWHAE	14-3-3 protein epsilon	255	29173.9	4.63	-0.54
GLO1	Lactoylglutathione lyase	183	20646.52	5.12	-0.529
PRKAR1A	cAMP-dependent protein kinase type I-alpha regulatory subunit	381	42981.69	5.27	-0.437

Supplementary Table S2. Efficiency of albumin removal per method

Sample preparation method	Albumin mean intensity	Albumin rank
IGD	3.55E+08	1
SP3	7.36E+08	1
IPA/TCA	3.69E+07	10
Top 14	1.26E+08	3
PreOmics	9.76E+07	11
Seer	2.75E+07	25

Supplementary Table S3. Endogenous concentrations of spiked-in proteins based on the data retrieved from Human Protein Atlas and the applied concentration range

Gene name	Protein Description	Detected by Immunoassays (ng/µl)	Detected by MS (ng/µl)	Working range (ng/µl)
CRP	C-reactive protein	2.9	3.9	0.5-4
S100A8	Protein S100-A8	0.011	0.27	0.5-4
LYZ	Lysozyme C	1.5	0.99	0.25-2
PPIA	Peptidyl-prolyl cis-trans isomerase A	-	0.21	0.25-2
YWHAE	14-3-3 protein epsilon	-	0.1	0.0625-0.5
GLO1	Lactoylglutathione lyase	-	0.0017	0.05-0.4
PRKAR1A	cAMP-dependent protein kinase type I-alpha regulatory subunit	-	0.0017	0.05-0.4

Supplementary Table S4. Endogenous concentrations (ng/µl) of spiked-in proteins after applying the standard addition method for each sample preparation method

Gene name	Protein Description	IGD	SP3	IPA/TCA	Top14	PreOmics	Seer
CRP	C-reactive protein	1.63	1.72	1.67	1.55	1.94	0.54
S100A8	Protein S100-A8	0.50	1.36	0.43	0.13	0.12	0.03
LYZ	Lysozyme C	0.86	1.01	1.31	0.67	0.75	0.54
PPIA	Peptidyl-prolyl cis-trans isomerase A	1.69	1.45	0.50	1.79	0.02	0.18
YWHAE	14-3-3 protein epsilon	-	-	-	-	-	0.05
GLO1	Lactoylglutathione lyase	-	-	-	-	-	-
PRKAR1A	cAMP-dependent protein kinase type I-alpha regulatory subunit	-	-	-	-	-	0.12

Supplementary Table S5. Number of identified stripped peptide IDs (without taking modifications and charge state into account) for spiked-in proteins across all methods

Gene name	Protein Description	IGD	SP3	IPA/TCA	Top 14	PreOmics	Seer
CRP	C-reactive protein	7	5	6	6	5	5
S100A8	Protein S100-A8	6	6	2	6	12	10
LYZ	Lysozyme C	5	7	2	6	7	5
PPIA	Peptidyl-prolyl cis-trans isomerase A	9	6	6	5	7	9
YWHAE	14-3-3 protein epsilon	0	0	0	1	0	14
GLO1	Lactoylglutathione lyase	1	1	1	0	5	10
PRKAR1A	cAMP-dependent protein kinase type I-alpha regulatory subunit	4	4	0	11	13	15
